# Carbonic Anhydrase Protects Fatty Liver Grafts against Ischemic Reperfusion Damage

**DOI:** 10.1371/journal.pone.0134499

**Published:** 2015-07-30

**Authors:** Mohamed Bejaoui, Eirini Pantazi, Viviana De Luca, Arnau Panisello, Emma Folch-Puy, Georgina Hotter, Clemente Capasso, Claudiu T. Supuran, Joan Rosselló-Catafau

**Affiliations:** 1 Department of Experimental Pathology, Institute of Biomedical Research of Barcelona-Spanish National Research Council (IIBB-CSIC), IDIBAPS, Barcelona, Spain; 2 Institute of Bioscience and Bioresources (IBBR), National Research Council, Napoli, Italy; 3 Neurofarba Department, University of Florence, Firenze, Italy; 4 Centro de Investigación Biomédica en Red de Enfermedades Hepáticas y Digestivas (CIBERehd), Barcelona, Spain; IDIBAPS - Hospital Clinic de Barcelona, SPAIN

## Abstract

Carbonic anhydrases (CAs) are ubiquitous metalloenzymes that catalyze the reversible hydration of carbon dioxide to bicarbonate and a proton. CAs are involved in numerous physiological and pathological processes, including acid-base homeostasis, electrolyte balance, oxygen delivery to tissues and nitric oxide generation. Given that these processes are found to be dysregulated during ischemia reperfusion injury (IRI), and taking into account the high vulnerability of steatotic livers to preservation injury, we hypothesized a new role for CA as a pharmacological agent able to protect against ischemic damage. Two different aspects of the role of CA II in fatty liver grafts preservation were evaluated: 1) the effect of its addition to Institut Georges Lopez (IGL-1) storage solution after cold ischemia; 2) and after 24h of cold storage followed by two hours of normothermic ex-vivo perfusion. In all cases, liver injury, CA II protein concentration, CA II mRNA levels and CA II activity were determined. In case of the *ex-vivo* perfusion, we further assessed liver function (bile production, bromosulfophthalein clearance) and Western blot analysis of phosphorylated adenosine monophosphate activated protein kinase (AMPK), mitogen activated protein kinases family (MAPKs) and endoplasmic reticulum stress (ERS) parameters (GRP78, PERK, IRE, eIF2α and ATF6). We found that CA II was downregulated after cold ischemia. The addition of bovine CA II to IGL-1 preservation solution efficiently protected steatotic liver against cold IRI. In the case of reperfusion, CA II protection was associated with better function, AMPK activation and the prevention of ERS and MAPKs activation. Interestingly, CA II supplementation was not associated with enhanced CO_2_ hydration. The results suggest that CA II modulation may be a promising target for fatty liver graft preservation.

## Introduction

Ischemia reperfusion injury (IRI) is the main cause of early allograft dysfunction after organ transplantation [1]. During liver graft storage in preservation solutions (cold ischemia), several complex processes occur, including ATP depletion, Ca^2+^ overload, cell swelling, cytoskeletal disruption, ion homeostasis dysregulation and acidosis, which all contribute to graft injury. Ischemic damage is exacerbated after graft revascularization due to oxygen entry and the subsequent generation of reactive oxygen species (ROS) [2].

The worldwide shortage of donor organs has led to the expansion of donor criteria to include suboptimal grafts such as steatotic livers, which in the past were excluded from transplantation [3] due to their association with primary non-function and delayed graft function [4]. It is well known that fatty livers are more vulnerable to IRI than non-fatty ones. This situation required the development of new alternatives to the University of Wisconsin preservation solution (UW), mainly used for abdominal organs, to improve both the conservation of these suboptimal liver grafts and the outcomes of transplantation [5, 6]. Recently, Institute Georges Lopez (IGL-1) solution has been shown to be a good alternative to the “gold standard” UW for fatty liver storage [7]. The benefits of IGL-1 are associated with the generation of nitric oxide (NO), a potent vasodilator agent which counterbalances the exacerbated microcirculation alterations present in steatotic liver grafts [8]. In addition, IGL-1 solution has been found to decrease endoplasmic reticulum stress (ERS) which leads to the inhibition of liver apoptosis [9].

Carbonic anhydrases (CAs) are ubiquitous zinc-metalloenzymes that catalyze the reversible hydration of carbon dioxide (CO_2_) forming bicarbonate (HCO_3_^-^) and a proton. CAs are present in many isoforms with different catalytic activity, tissues/organs distribution and subcellular localization [10]. CAs are involved in many pathophysiological processes such as pH and CO_2_ homeostasis, CO_2_ and HCO3^-^ transport between metabolizing tissues and lungs, electrolyte secretion, biosynthetic reaction (lipogenesis, ureagenesis and gluconeogenesis) and tumor cell survival [11]. Many of CAs biological functions cross-link with processes that occur in IRI, such as acid-base homeostasis, electrolyte balance, oxygen delivery to tissues [10] and nitric oxide generation/chemical modification (i.e., through its hydration) [12].

Several CA isoforms, including CA II, CA III and CA IX, have been described in liver (13,14, 15), but their relevance in the pathophysiology of IRI has not been adequately investigated to date. Recently, Carini *et al*. associated CA IX with the protective effects of liver hypoxic preconditioning, since it contributed to increasing hepatocyte tolerance against ischemia by maintaining intracellular Na^+^ homeostasis [13]. It has also been shown that CA IX inhibition may be useful for the management of hypoxic tumors resistant to classical chemotherapy and radiotherapy [14–16].

The objective of our study was to investigate the relevance of CA II in steatotic liver graft preservation. We explored whether the addition of CA II to IGL-1 preservation solution could protect steatotic liver grafts against cold IRI, as well as the potential underlying mechanisms. Our results shed new light on the mechanism of action of CA II and may contribute to the design of new effective strategies for fatty liver graft preservation.

## Materials and Methods

### Animals

Male homozygous obese Zucker rats, aged 9 to 10 weeks, were purchased from Charles River (France) and housed in a temperature-controlled environment (25°C) with a 12-hour light-dark cycle and provided water and standard chow *ad libitum*. In this study, animals underwent isolated liver perfusion (described further below). All experiments were approved by the Ethics Committee for Animal Experimentation (CEEA, Directive 697/14), University of Barcelona, and were conducted in accordance with European Union regulations for animal experiments (Directive 86/609 CEE).

### Liver procurement and *ex-vivo* perfusion

Obese rats were anesthetized under isoflurane inhalation. The surgical technique was performed as previously described [17]. Briefly, after cannulation of the common bile duct, livers were flushed with chilled preservation solution (4°C) by means of a catheter inserted into the aorta. After cooling, a second catheter was inserted into the portal vein to complete liver rinsing and the whole liver was excised and trimmed of surrounding tissues. During liver perfusion, animals were sacrificed by exsanguination under isoflurane inhalation. Forty milliliters of preservation solution were infused through the aorta and the portal vein. Then, the livers were preserved with a further 130 ml of the same solution for 24h at 4°C. Control livers (without preservation) were perfused with the oxygenated perfusion medium (described below) at 37°C immediately after harvesting. The cold storage solution used was IGL-1 solution, either supplemented or not with bovine CA II, at 10 μg/ml (Sigma Aldrich, Barcelona, Spain).

All fatty livers were perfused at 37°C via the portal vein in a closed, controlled pressure circuit. Time point 0 was established when the portal catheter was satisfactorily connected to the circuit. During the first 15 minutes of perfusion (the initial equilibration period), the flow was progressively increased in order to stabilize the portal pressure at 12mm Hg (Pression Monitor BP-1; Pression Instruments, Sarasota, FL). The flow was controlled by a peristaltic pump (Minipuls 3; Gilson, France). The reperfusion liquid (150 ml for each perfusion) consisted of a cell culture medium (William’s medium E; BioWhittaker, Barcelona, Spain) with a Krebs-Henseleit-like electrolyte composition enriched with 5% albumin as oncotic supply. The medium was continuously gassed with 95% O_2_ and 5% CO2 gas mixture and subsequently passed through a heat exchanger (37°C) and a bubble trap prior to entering the liver. After 120 minutes of normothermic reperfusion, the effluent perfusion fluid was collected for biochemical determination, and fatty livers were sampled.

### Experimental groups

All male obese Zücker rats were randomized according to the following experimental protocols:

#### Protocol 1: Cold ischemia

1. Control group (Ctr 1): After laparotomy, blood was collected and immediately centrifuged for 10 min at 3000 g for transaminase assay, and liver samples were collected and stored at -80°C for further determinations (n = 4).
2. IGL-1 group: Livers from 5 Zucker Ob rats were flushed with IGL-1 preservation solution and preserved for 24h at 4ªC in the same solution. Livers were then flushed with Ringer’s lactate solution at room temperature *via* the portal vein and sampled. Aliquots of the effluent flush were collected for measurement of cumulative transaminases after 24 h cold storage.
3. IGL-1+CAII group: The same as group 2, but fatty livers were flushed and then preserved in IGL-1 solution supplemented with bovine CA II at 10 ug/ml (n = 5).

#### Protocol 2: Cold ischemia and reperfusion

1. Control group (Ctr 2) (n = 4): After procurement, steatotic livers were ex-vivo perfused for 2h as described above without prior cold storage.
2. IGL-1 group (n = 6): Fatty livers were preserved in IGL-1 preservation solution for 24 hours at 4°C and then subjected to 2h of normothermic reperfusion at 37°C.
3. IGL-1+CAII (n = 6): Fatty livers were preserved in IGL-1 supplemented with bovine CA II at 10 ug/ml and then ex-vivo perfused for 2 hours at 37°C.

### Hepatic damage: AST/ALT and histology

Hepatic injury was assessed on the basis of alanine aminotransferase (ALT) and aspartate aminotransferase (AST) levels with commercial kits from RAL (Barcelona, Spain). Briefly, 100 μL of effluent perfusate were added to 1 ml of the substrate provided by the commercial kit, and transaminase activity was then measured at 340 nm with an UV spectrometer and calculated following the supplier’s instructions. Results were normalized using a commercial calibrator (Biocal, RAL, Barcelona, Spain)

Liver samples were fixed in 10% neutral buffered formalin and embedded in Paraplast, and 5-μm sections were stained with hematoxylin and eosin according to standard procedures. Histological evaluation was graded semi quantitatively from 0 (no damage) to 4 (severe cellular damage, such as vacuolization, cell dissociation, cell swelling and disintegration of the hepatic architecture).

### Bile output and hepatic clearance

Liver function was assessed by the measurement of bile production. Bile was collected through the cannulated bile duct, and the output (μL of bile/g of liver) was reported.

Hepatic clearance was considered another parameter of hepatic function. Thirty minutes after the onset of perfusion, 10 mg of bromosulfophthalein (BSP) (Sigma, Madrid, Spain) was added to the perfusate. The concentration of BSP in the bile samples after two hours of normothermic perfusion was measured at 580 nm with an ultraviolet-visible spectrometer. Bile BSP excretion was expressed as a percentage of the perfusate content (t_120_ of bile /t_30_ of perfusate × 100)

### ATP quantitation

ATP was extracted as previously described using phenol-TE extraction [18]. Then ATP concentration was measured using a firefly bioluminescence assay kit (ATP Determination Kit A22066, Life Technology, Madrid, Spain) according to supplier’s instructions. Briefly, ATP extract was diluted 1000-fold and then 10 μL was injected into 90 μL of luciferase reagent and measured immediately in a luminometer (Orion Microplate Luminometer, Berthold detection system, Pforzheim, Germany). A calibration curve with dilutions of ATP from 1.25 10^−6^ to 10^−7^ was also prepared. Data were normalized as ATP μmol g^-1^ wet tissue.

### Real-time qRT-PCR

Total liver RNA was isolated using the TRIzol reagent (Invitrogen). Reverse transcription was performed on a 1 μg RNA sample using the iScript cDNA Synthesis Kit (Bio-Rad Laboratories). The reaction included incubation at 25°C (5 min), at B°C (30 min) and 85°C (5 min), and then cDNA was stored at -80°C. Subsequent PCR amplification was conducted in the iCycler iQ Multi-Color Real-Time PCR (Bio-Rad Laboratories) using SsoAdvanced Universal SYBR Green Supermix and the following rat primers for CA II: forward, 5'-TGGTTCACTGGAACACCAAA-3' and reverse, 3'-AAAACTTCTAACCTGGACGG-5´. Reactions were carried out in duplicate and threshold cycle values were normalized to glyceraldehyde-3-phosphate dehydrogenase (GAPDH) gene expression. The ratio of CA II relative expression to GAPDH was calculated by the ΔCt formula.

### Western blotting analysis

Liver tissue was homogenized in HEPES buffer, and proteins were separated by SDS-PAGE and transferred to PVDF membranes. Membranes were immunoblotted using the following antibodies: anti-GRP78 (GRP78 (H-129): sc-13968, Santa Cruz Biotechnology Inc, CA, USA); anti-carbonic anhydrase II (ab115306, abcam, UK), anti-ATF6 (ab11909, abcam, UK), anti-p-AMPKα (Thr172, #2535); anti-AMPKα (#2603), anti-cleaved Caspase-3 (Asp175, #9664), anti-p-eIF2α (Ser51, #9721), anti-IRE1α (#3294), anti-p-PERK (Thr980, #3179), anti-p-SARK/JNK (Thr183/Tyr185), anti-p-p38 MAP kinase (Thr180/Tyr182, #9211), anti-p-p44/42 MAPK (Erk1/2, Thr202/Tyr204, #9101); the above antibodies were all purchased from Cell Signaling (Danvers, MA), anti-eNOS (610296, from Transduction Laboratories, Lexington KY), and anti-b-actin (A5316, Sigma Chemical, St. Louis, MO, USA). After washing, bound antibody was detected after incubation for 1 h at room temperature with the corresponding secondary antibody linked to horseradish peroxidase. Bound complexes were detected and quantified by scanning densitometry.

### Immunohistochemistry

Antibodies for immunohistochemical analysis were as follows: mouse monoclonal anti-CA II (sc-48351; Santa Cruz, Barcelona, Spain) at a dilution of 1:50 and rabbit polyclonal anti-CHOP (GADD 153 (F168): sc575; Santa Cruz, Barcelona, Spain) at a dilution of 1:100. Liver samples were fixed and embedded in paraffin. Antigen retrieval was performed by incubating samples with 10 mM sodium citrate. After blocking, the tissue sections were incubated overnight with the antibody. Sections were then incubated with biotinylated goat anti-mouse IgG (1:200) for CA II or with biotinylated goat anti rabbit IgG (1:200) for CHOP followed by conjugated horseradish peroxidase-streptavidin (VECTASTAIN Elite ABC Reagent). Slides were counterstained with hematoxylin and mounted with DPX (Sigma, Madrid, Spain). Images were taken with Nikon Eclipse E1000 microscope (Nikon, Lewisville, TX) using Cell B imaging software (Olympus America, Center Valley, PA; USA).

### TUNEL staining

Apoptosis was determined by *in situ* detection of DNA fragmentation using terminal deoxynucleotidyl transferase-mediated 29-deoxyuridine 59-triphosphate nick end labeling (TUNEL) assay with the *in situ* apoptosis detection kit (Click-iT Plus TUNEL Assay, Life Technology, Madrid). TUNEL staining was performed according to the manufacturer’s instruction in paraffin sections of 6 μm thickness. Then the sections were stained with DAPI. The sections were examined with fluorescence microscopy (Nikon, Lewisville, TX).

### Carbonic anhydrase activity

#### Sample preparation

Livers from all samples (approximately, 300 mg) were homogenized in 0.05 M Tris-HCl buffer, pH 7.5. The homogenate was centrifuged twice for 30 min at 12000*xg*. The resulting supernatant was subjected to protein determination and assayed for CO_2_ hydratase activity.

#### Protein determination

Protein concentration was determined using the Bio-Rad Protein Assay, based on the Bradford method [19].

#### Carbonic Anhydrase assay

CA activity assay was a modification of the procedure described by Chirica et al [20]. The assay was based on the monitoring of pH variation due to the catalyzed conversion of CO_2_ to bicarbonate. Bromothymol blue was used as the indicator of pH variation. The assay was performed at 0°C adding 1.0 mL ice-cold CO_2_-saturated water to 1.0 mL mixtures of 25 mM Tris-SO4 buffer. The CO_2_-saturated solution was prepared by bubbling CO_2_ into 100 mL distilled water for approximately 3 h. The CO_2_ solution was chilled in an ice water bath. 50 μL of the liver extract were added to one tube, and an equivalent amount of buffer was added to the second tube as control. One milliliter of CO_2_ solution was added very quickly, and simultaneously a stopwatch was started. The time required for the solution to change from blue to yellow was recorded (transition point of bromothymol blue is pH 6–7). The production of hydrogen ions during the CO_2_ hydration reaction lowers the pH of the solution until the color transition point of the dye is reached. The time required for the color change is inversely related to the quantity of carbonic anhydrase present in the sample. Detecting the color change is somewhat subjective but the error for triple measurements was within the range of 0–1 s difference for the catalyzed reaction. Wilbur-Anderson units were calculated according to the following definition: one Wilbur-Anderson unit (WAU) of activity is defined as (T_0_ − T)/T, where T_0_ (uncatalyzed reaction) and T (catalyzed reaction) are recorded as the time (in seconds) required for the pH to fall from 8.3 to the transition point of the dye in a control buffer and in the presence of the enzyme respectively.

### Statistical analysis

Data are expressed as means ± SE and compared statistically by variance analysis, followed by the Student-Newman-Keuls test (Graph Pad Prism software). *P* < 0.05 was considered significant.

## Results

### Protocol 1: Cold ischemia

#### Effect of CA II on steatotic liver injury after cold ischemia

**Fig 1:**
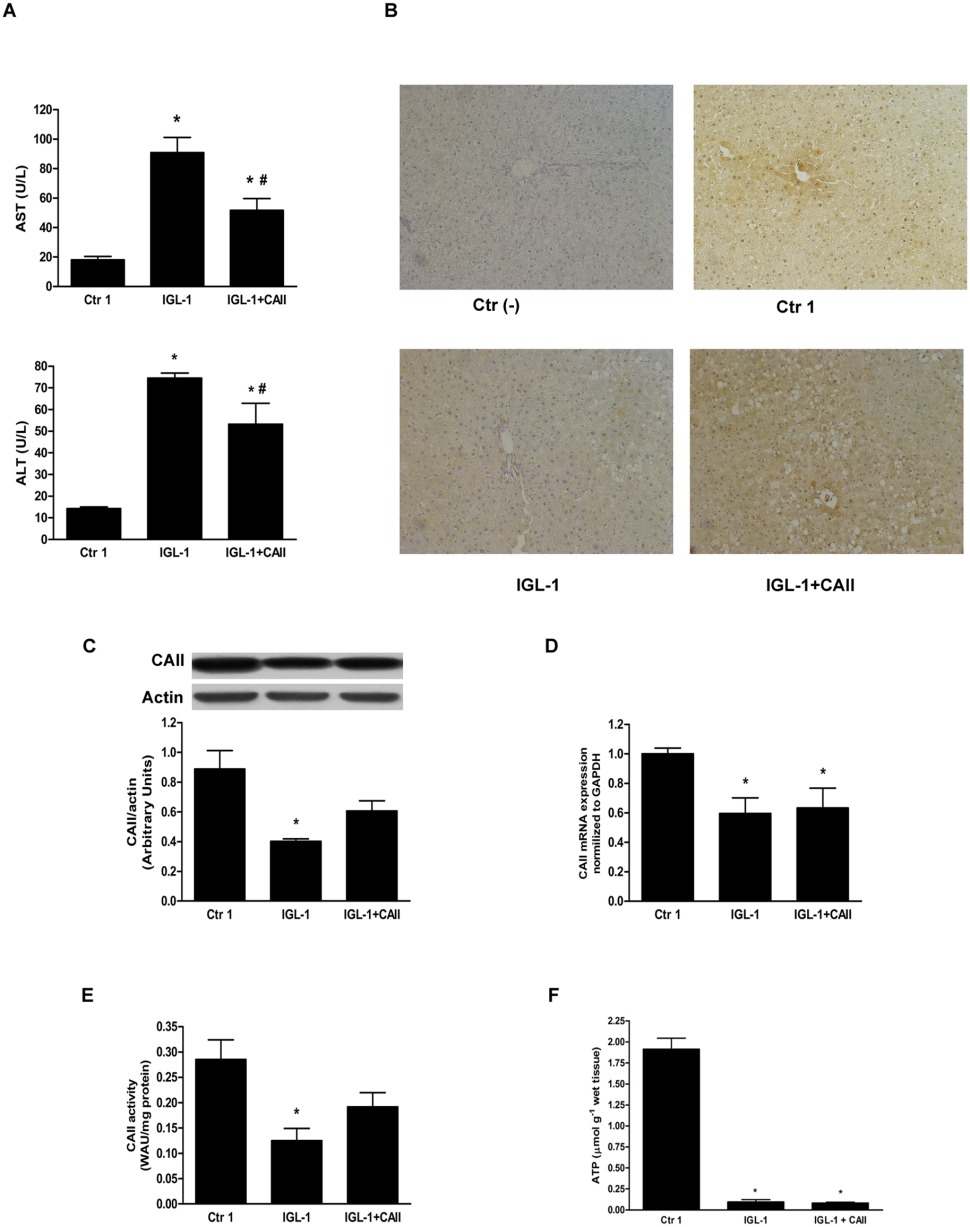
Hepatic injury and CA II expression and activity after cold ischemia. (A) Hepatic injury after cold ischemia in IGL-1 solution supplemented or not with CA II, measured as AST and ALT levels. The presence of CA II in IGL-1 significantly reduced AST/ALT levels; (B) CA II immunohistochemistry: CA II staining was reduced after 24h-cold storage in IGL-1 solution (IGL-1). CA II staining increased in IGL-1+CAII group compared to IGL-1 group; (C) CA II protein expression by Western blotting and densitometric analyses. A fall in CA II expression was observed after 24 h-cold ischemia in IGL-1 solution, which was slightly reversed after CA II supplementation of IGL-1 solution; (D) Quantitative CA II mRNA expression in steatotic livers preserved in IGL-1 or IGL-1+CAII. CA II mRNA levels presented similar reductions in both IGL-1 solutions compared to controls; (E) CA II activity levels in fatty livers preserved in IGL-1 or IGL-1+CAII. CA II activity decreased in livers preserved in IGL-1 solutions compared to controls; (F) ATP quantitation: ATP levels decrease significantly after cold storage. Ctr 1: liver flushed without cold preservation; IGL-1: liver preserved in IGL-1 solution; IGL-1+CAII: liver preserved in IGL-1 solution enriched with CA II. * p < 0.05 vs Ctr 1; # p < 0.05 vs IGL-1.

Transaminase levels were measured in the effluent of washout liquid (Ringer’s lactate) after cold storage. Livers preserved in IGL-1 preservation solution showed high ALT and AST levels, whereas the addition of CA II to IGL-1 solution (IGL-1+CAII) significantly reduced transaminase levels (Fig 1A). Next, we studied the CA II profiles obtained by immunohistochemistry and Western blot techniques. Livers preserved in IGL-1 solution for 24 hours showed a significant decrease in CA II staining when compared to control livers (Fig 1B). This situation was reversed by CA II addition to IGL-1 solution, as revealed by the increased CA II staining in the IGL+CAII group. These results agreed with those obtained by Western blot analysis. In fact, CA II protein expression decreased significantly after cold storage and increased after the addition of CA II to IGL-1 solution (Fig 1C). Next, we examined the mRNA levels of CA II (Fig 1D). We found that CA II mRNA expression decreased after cold storage and the addition of CA II did not affect mRNA expression. Finally, we analyzed CA II activity and observed that preservation of fatty livers in IGL-1 solution resulted in decreased CA II activity, which slightly increased after CA II addition (Fig 1E). These results were consistent with the Western blot and immunochemistry findings. Finally, we assessed ATP content in fatty liver submitted to prolonged cold storage. As expected, cold ischemia was associated with marked decrease in liver ATP content and addition of bovine CA II did not enhance ATP level.

### Protocol 2: Cold ischemia and reperfusion

#### Effect of CA II on steatotic liver injury after normothermic reperfusion

**Fig 2:**
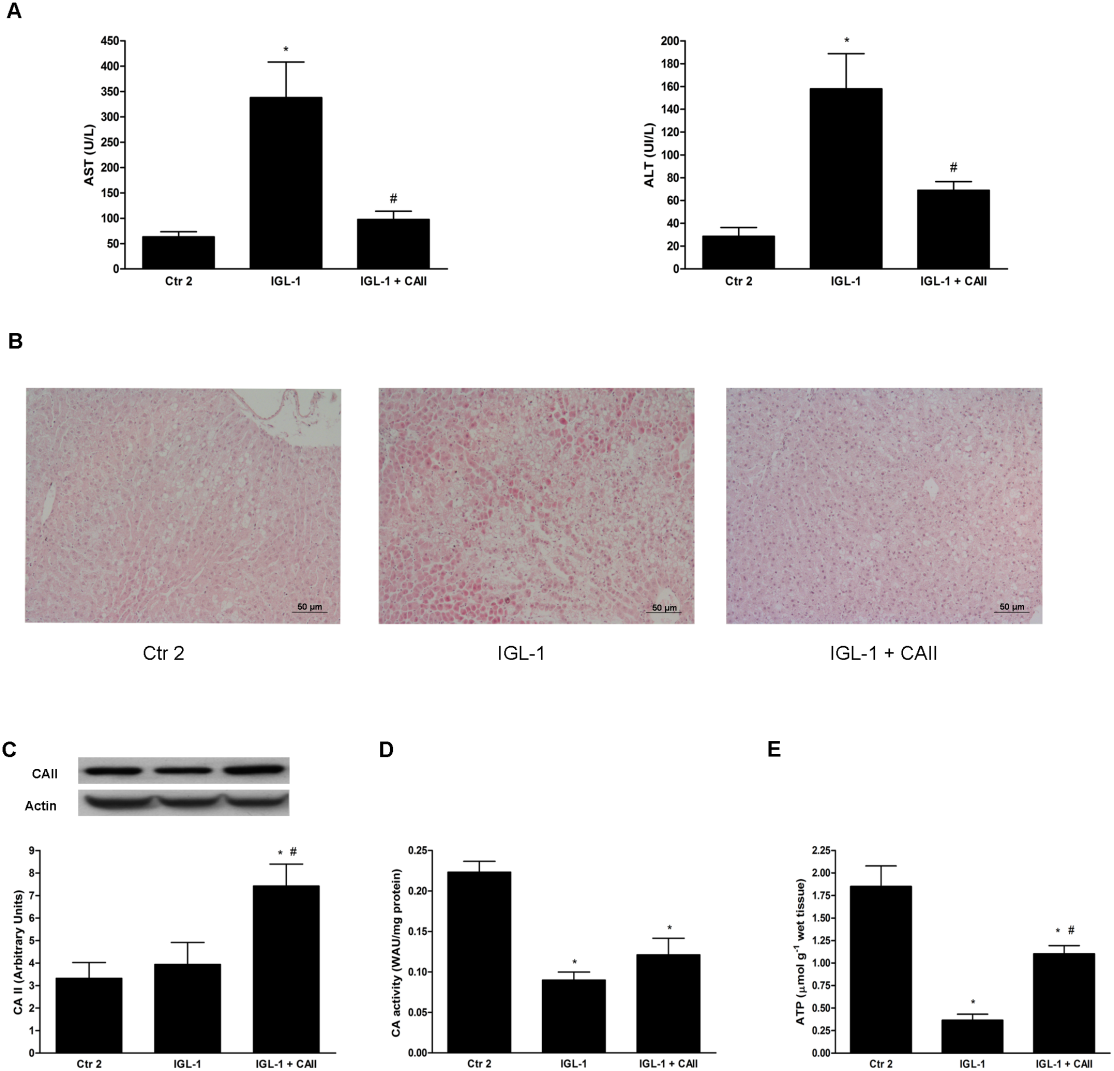
Hepatic injury and CA II expression and activity after cold ischemia and normothermic reperfusion. (A) Hepatic injury measured as AST and ALT levels after 2h-normotermic *ex-vivo* perfusion. CA II supplementation of IGL-1 attenuated AST/ALT levels, in contrast to the IGL-1 group. (B) Hematoxylin and eosin staining. The IGL-1 group presented increased hepatic damage compared to the control group (Ctr 2), while CA II addition to IGL-1 diminished hepatic damage compared to the IGL-1 group; (C) CA II protein expression by Western blot analyses. CA II expression was increased in the IGL-1+CAII group compared to Ctr 2 and IGL-1 groups; (D) CA II activity levels in fatty livers after 2h-normothermic reperfusion. CA II activity levels decreased in livers preserved in IGL-1 and IGL-1+CAII groups when compared to the control group (Ctr 2); ATP quantitation: ATP levels was partially restored in liver preserved in IGL-1 supplemented with CA II. Ctr 2: Livers flushed and perfused *ex-vivo* without cold preservation; IGL-1: liver preserved in IGL-1 solution (4°C, 24 h) and then subjected to 2h-normothermic *ex vivo* perfusion. IGL-1+CAII: liver preserved in IGL-1 solution (4°C, 24 h) enriched with CA II and subjected to 2h-normothermic *ex vivo* perfusion * p < 0.05 vs Ctr 2; # p < 0.05 vs IGL-1.

Addition of CA II to IGL-1 solution significantly decreased AST/ALT levels after 2 hours of normothermic perfusion when compared to IGL-1 group (Fig 2A).

These data were corroborated by the histological findings (Fig 2B). Livers preserved in IGL-1 group demonstrated severe cellular damage which was prevented by the presence of CA II; in the IGL-1+CAII group, hepatocyte integrity was similar to that of the control group. Interestingly, livers preserved in IGL-1+CAII exhibited a drastic reduction in fat infiltration when compared to the other experimental groups.

Next, we assessed CA II expression and activity (Fig 2C and 2D, respectively), finding that CA II protein levels increased in the livers preserved in IGL-1+CAII preservation solution compared to both control and IGL-1 groups. This increase was not observed when an anti-CA II antibody that did not recognize bovine CA II was used (data not shown). This finding indicates that the CA II protein increase recorded is due to the added bovine CA II. Conversely, the increase in CA II expression was not concomitant with an enhancement in CA II activity. Moreover, our data show that CA hydratase activity decreased significantly in both IGL-1 and IGL-1+CAII groups. Also, we measured ATP content after normothermic reperfusion. ATP recovery after two hours of reperfusion did not rich physiological levels as we observed a significant reduction in ATP levels in preserved livers when compared to control ones. Remarkably, livers preserved in IGL-1 solution enriched with CA II show a significant higher levels of ATP content than livers preserved in IGL-1 solution alone (Fig 2E).

#### Effect of CA II on hepatic function after normothermic reperfusion

**Fig 3:**
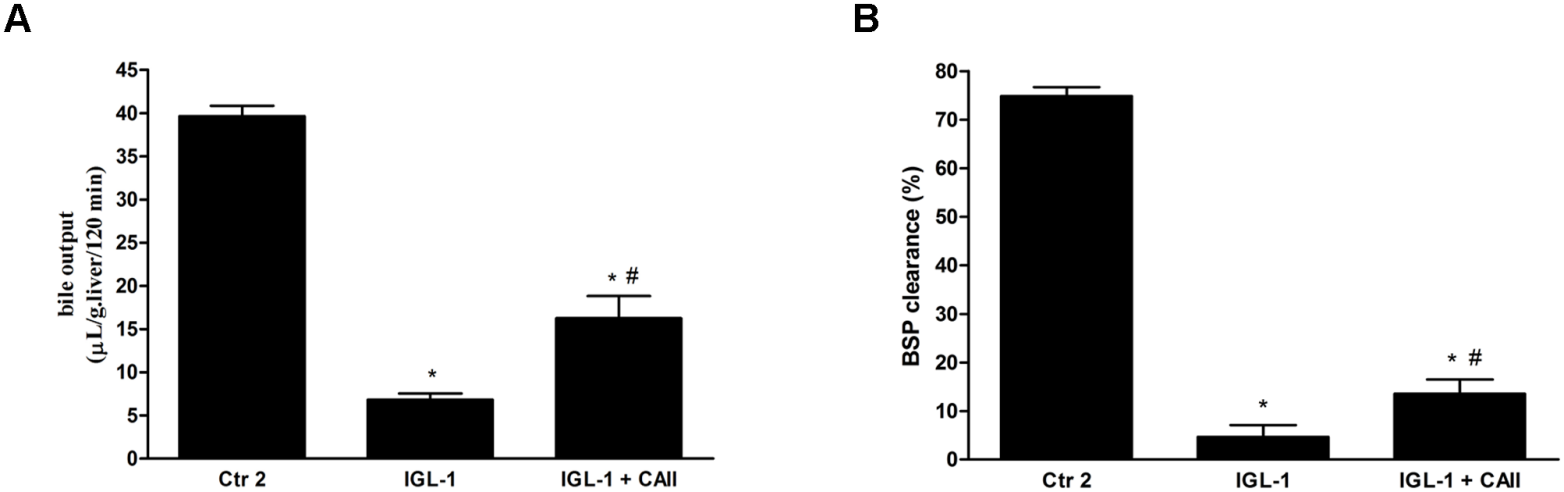
Effect of CA II addition to IGL-1 solution in hepatic function. Hepatic function expressed as bile output (A) and percentage of bromosulfophthalein (BSP) excretion in bile (B) after 120 min of fatty liver normothermic *ex vivo*perfusion. CA II supplementation (IGL-1+CAII) significantly enhanced bile production and BSP clearance compared to the IGL-1 group. Ctr 2: Livers flushed and perfused *ex-vivo* without cold preservation; IGL-1: liver preserved in IGL-1 solution (4°C, 24 h) and subjected to 2h-normothermic *ex vivo* perfusion. IGL-1+CAII: livers preserved in IGL-1 solution (4°C, 24 h) enriched with CA II (10 ug/ml) and subjected to 2h- normothermic *ex vivo* perfusion; * p < 0.05 vs Ctr 2; # p < 0.05 vs IGL-1.

Furthermore, we evaluated the liver function by measuring bile production and BSP clearance after two hours of reperfusion. Significant reductions in these parameters were observed in fatty livers preserved in IGL-1 solution in comparison with the control group (Fig 3A and 3B respectively). However, both liver function parameters improved significantly when CA II was added to IGL-1 solution (Fig 3).

#### Effect of CA II on AMPK activation and endoplasmic reticulum stress after normothermic reperfusion

**Fig 4:**
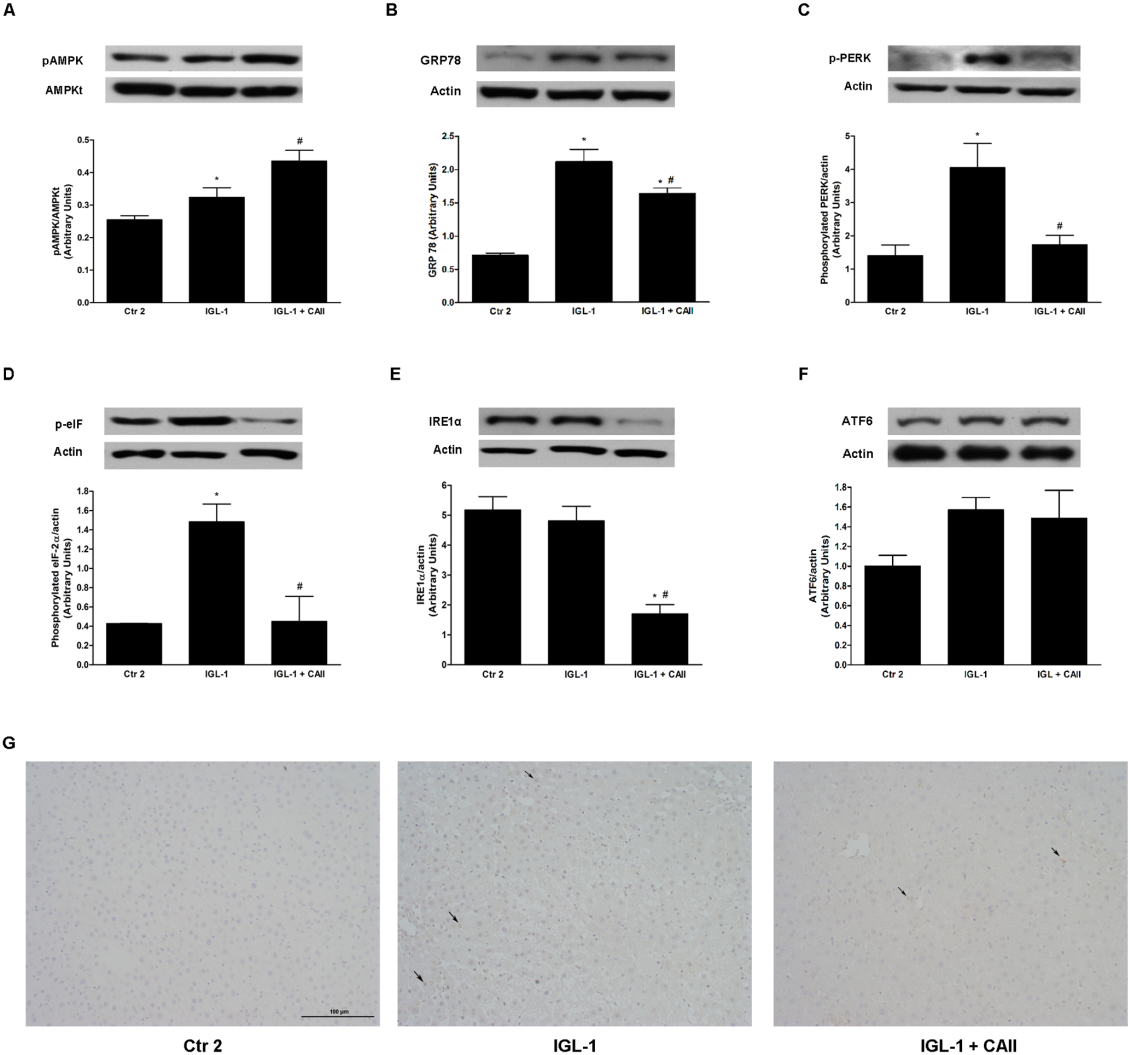
Effect of CA II addition to IGL-1 solution on AMPK activation and endoplasmic reticulum stress (ERS). Western blotting and densitometric analysis of pAMPK (A); GRP78 (B); p-PERK (C); p-eIF (D); IRE1alpha (E) and ATF6 (F) and immunohistochemistry of CHOP (G) in steatotic liver after 120 min of normothermic *ex vivo* perfusion. CA II supplementation (IGL-1+CAII) promoted AMPK activation and induced significant decreases in ERS parameters Without affecting CHOP expression. Ctr2: Livers flushed and perfused *ex-vivo* without cold preservation; IGL-1: livers preserved in IGL-1 solution (4°C, 24 h) and subjected to 2h-normothermic *ex vivo* perfusion. IGL-1+CAII: liver preserved in IGL-1 solution (4°C, 24 h) enriched with CA II and subjected to 2h-normothermic *ex vivo* perfusion. * p < 0.05 vs Ctr 2; # p < 0.05 vs IGL-1.

AMPK is a well-known cytoprotective factor which is induced in ischemic conditions in order to cease the metabolic pathways that consume ATP and to promote the ones that generate more ATP [21]. For this reason, we further determined pAMPK protein levels after reperfusion. We observed that IGL-1 supplementation with CA II provoked pAMPK increases in comparison to IGL-1 group (Fig 4A).

**Fig 5:**
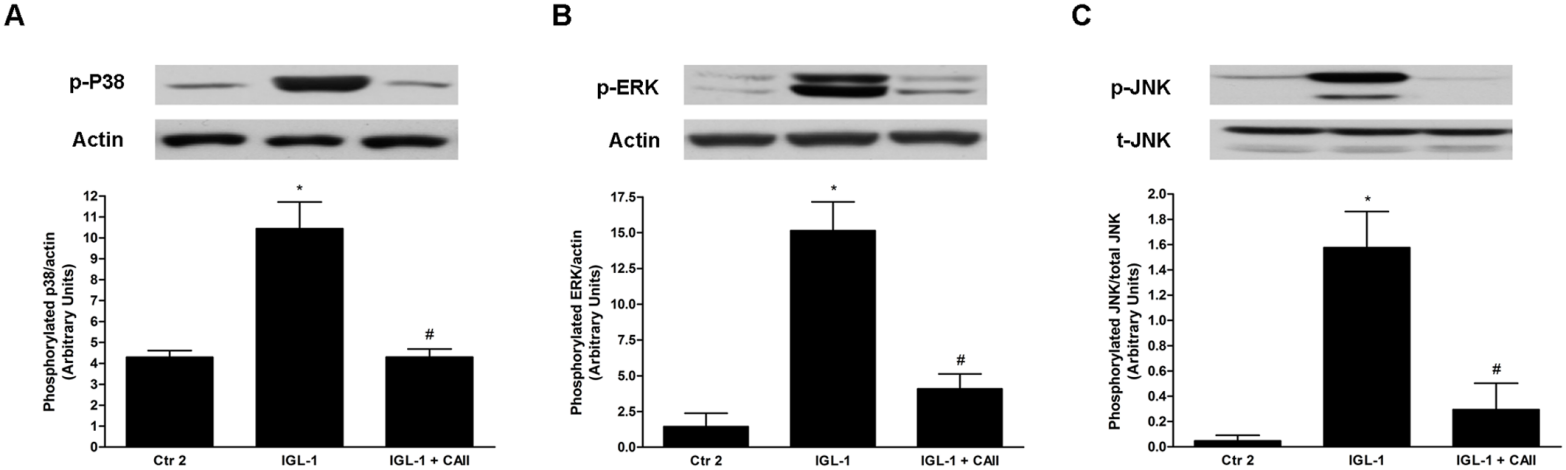
MAPK activation changes after enrichment of IGL-1 solution with CA II. Western blotting and densitometric analysis of p-38 (A); p-ERK (B) and p-JNK (C) in steatotic liver after 120 min of normothermic “ex vivo” perfusion. CA II addition to IGL-1 solution prevented MAPK activation. Ctr 2: Liver flushed and perfused “ex-vivo” without cold preservation; IGL-1: liver preserved in IGL-1 solution (4°C, 24 h) and subjected to 2h-normothermic “ex vivo” perfusion. IGL-1+CAII: liver preserved in IGL-1 solution (4°C, 24 h) enriched with CA II and subjected to 2h- normothermic “ex vivo” perfusion. * p < 0.05 vs Ctr 2; # p < 0.05 vs IGL-1.

Since AMPK activation can affect ERS, a common characteristic of IRI [22–24], we further examined glucose-regulated/binding immunoglobulin protein (GRP78) which is known to be induced by ERS. We found that GRP78 was upregulated in IGL-1 group when compared to control group and that supplementation of IGL-1 with CA II resulted in attenuation of GRP78. Then, we examined signaling pathways of the unfolded protein response (UPR) involved in CA II protection. Our results show that CAII significantly decrease PKR-like ER kinase (PERK), inositol-requiring enzyme 1 alpha (IRE1α) and eukaryotic translation initiation factor 2 (eIF2α) activation (Fig 5B, 5C and 5D respectively). Remarkably, transcription factor 6 (ATF6) did not result in a significant activation after ischemia reperfusion (Fig 5E). It is well-known that ERS is capable to induce an apoptogenic program through activation of DNA damage-inducible gene 153/C/EBP homologous protein (GADD153/CHOP). For this reason, we assessed CHOP profile by immunohistochemistry. Interestingly, no differences between the three groups were found (Fig 5F).

#### Effect of CA II on MAPKs activation after normothermic reperfusion

It is well known that during cold ischemia, MAPKs are activated and induce cellular injury through apoptosis and inflammation activation [25]. In our conditions, we observed that preservation of fatty livers in IGL-1 solution caused a major increase in MAPK protein expression (p-p38, pERK, pJNK), which was reversed by the addition of CA II to IGL-1 (Fig 5).

#### Effect of CA II on liver apoptosis after normothermic reperfusion

**Fig 6:**
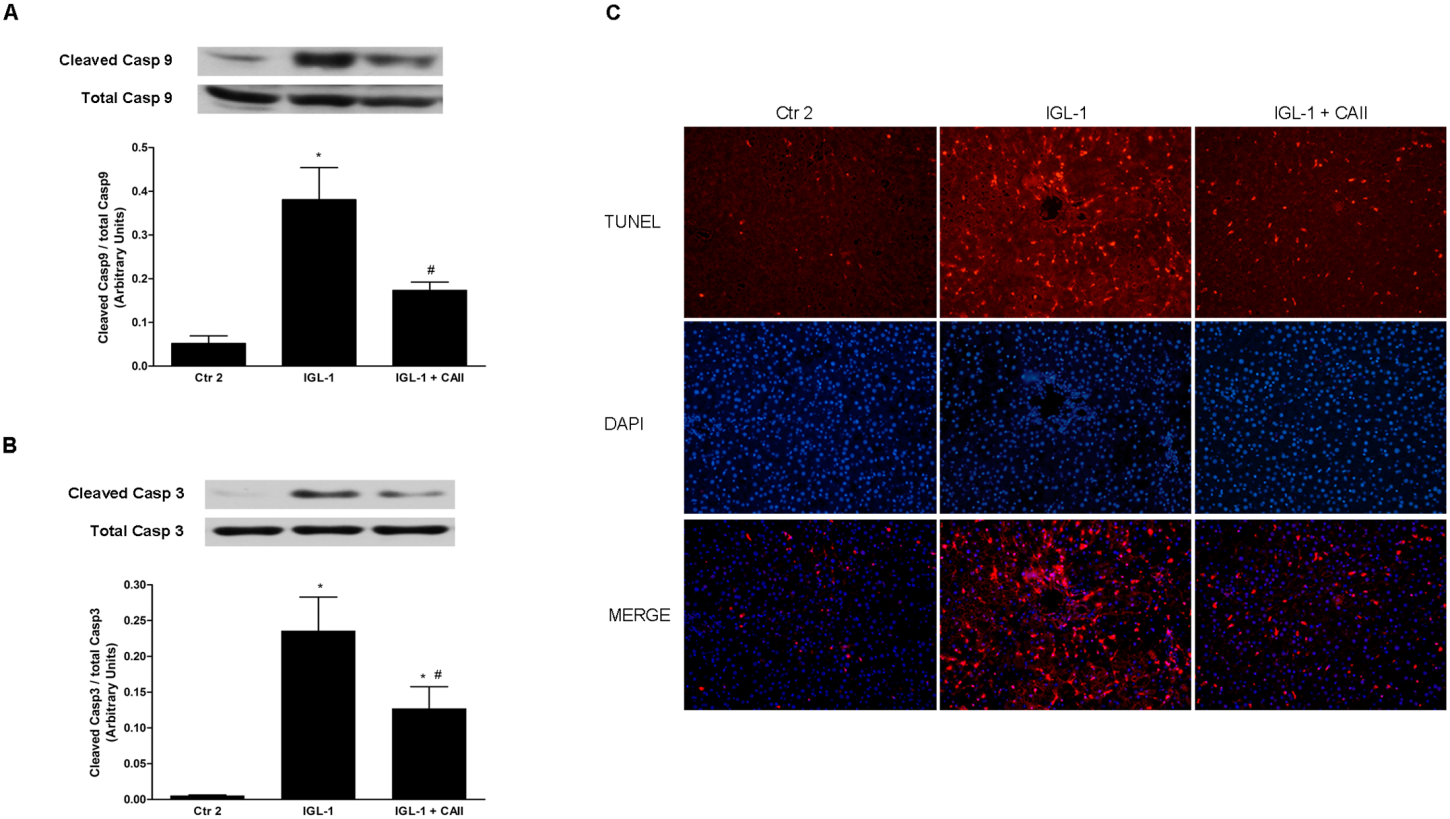
Effects of CA II addition to IGL-1 solution in liver apoptosis. Liver apoptosis, measured as cleaved caspase 9/ total caspases 9 (A), cleaved caspase 3/ total caspase 3 (B) and TUNEL assay (C) in steatotic livers after 120 min of normothermic “ex vivo” perfusion. CA II addition to IGL-1 solution reduced activation of liver caspases 3 and 9 and diminished TUNEL-positive cells. Ctr 2: Liver flushed and perfused “ex-vivo” without cold preservation; IGL-1: liver preserved in IGL-1 solution (4°C, 24 h) and subjected to 2h- normothermic “ex vivo” perfusion. IGL-1+CAII: liver preserved in IGL-1 solution (4°C, 24 h) enriched with CA II and subjected to 2h-normothermic “ex vivo” perfusion. * p < 0.05 vs Ctr 2; # p < 0.05 vs IGL-1.

IRI is also associated with the initiation of apoptotic pathways [25]. In our conditions, we observed that preservation of fatty livers in IGL-1 solution supplemented by CA II caused a substantial decrease in liver apoptosis, as reflected by reductions in cleaved caspases 9 and 3 protein expression and TUNEL staining (Fig 6A, 6B and 6D respectively).

## Discussion

In the present study, we observed that hepatic injury in fatty livers during cold storage in IGL-1 solution is associated with reduced levels of CA II expression and activity. We therefore hypothesized that CA II addition to IGL-1 solution could protect fatty livers against ischemic damage during cold storage. Indeed, our results demonstrated that the addition of bovine CA II to IGL-1 preservation solution improved steatotic liver preservation in cold ischemia by enhancing the expression and the activity of CA II in liver tissue, thus acting as a “liver protector” against IRI.

The rational of using CA II in preservation solution was the similarities between the events occurring during ischemia and CAs physiological and pathological processes such as acid-base homeostasis, electrolyte balance, oxygen delivery to tissues and nitric oxide generation. Also, we focused our study in fatty livers because of their high vulnerability to IRI. However, whether CA II is associated with fatty liver or not is still to be investigated. It has been reported that cytosolic CA II is necessary for the *de novo* lipid synthesis pathway and more specifically for malonyl-CoA formation. Thus, CA II inhibitor has been suggested as antiobesity drugs [26]. We have also tested the effect of CA II addition to IGL-1 solution in lean Zucker rats and we failed to observe any protective effect (data not shown), suggesting a close relationship between CA modulation and obesity. However we cannot exclude that the protective effects against IRI in non-obese rats could be achieved at different concentrations.

The principle of static cold storage is the reduction of metabolism and oxygen requirements by hypothermia, in order to prevent tissue injury [5]. As described in the literature, the addition of testicular hyaluronidase, an enzyme responsible for the degradation of hyaluronane, to UW preservation solution limits liver cell damage and improves graft function after orthotopic liver transplantation [27]. These data confirm that the addition of an enzyme to preservation solutions may have a protective effect on organ preservation even at low temperature. Moreover, CA activity was assayed at 0°C using the colorimetric method, suggesting that CA shows significant activity at low temperatures.

In the present paper, we also investigated whether CA II might be beneficial in the prevention of reperfusion injury. Our results demonstrated that CA II addition made an effective contribution to the protection of steatotic livers against ischemic-reperfusion insult, as evidenced by the decrease in transaminase levels and the histological findings. The protective effect in liver injury was associated with a better liver function when they were preserved in the IGL-1+CAII preservation solution. In fact, this group, presented higher increases in bile production and BSP clearance.

The observed protection was associated with a significant increase in CA II protein levels which was attributed to the exogenous bovine CA II. Interestingly, supplementation of CA II did not lead to an increase in its activity. For these reasons, we may speculate that the protective mechanism of CA II in this scenario is related to a direct interaction of this enzyme with some receptors or even to non-hydratase activity of CA II. Indeed, CA II has shown to produce nitric oxide through nitrite reduction and this reaction was enhanced at acidic pH like that occurring during cold storage [12].

AMPK is a metabolic sensor activated in case of decreased energy status in order to switch off pathways that consume ATP which lead toward an energy conserving state [21]. Indeed, enrichment of UW solution with an AMPK activator, 5-amino-4-imidazole carboxamide riboside (AICAR), ameliorated fatty liver cold storage [28]. In this study, CA II addition to IGL-1 solution activated AMPK which was consistent with increased ATP levels found at reperfusion.

Recently, a close relationship between AMPK and ERS has been described. AMPK activation reduced IRI through ERS reduction in a model of cardiac hypoxia reoxygenation [23] and in an isolated perfused steatotic rat liver model [24]. It has also been shown that ERS contributed to the poor tolerance of steatotic livers to IRI and mediated post-transplant injury and cell death [22, 29]. In accordance with these data, our results show that activation of AMPK was consistent with the reduction in ERS parameters. CA II supplementation to IGL-1 solution inhibited PERK and IRE1 pathway; however ATF6 pathway was not significantly activated after two hours of reperfusion. Additionally, we examined ERS induced apoptosis through CHOP assessment and we found no significant differences in its protein expression pattern. We may suggest that the 2-hours duration of reperfusion is not sufficient to enhance CHOP expression. Although in the literature it is very well reported the expression of ER signaling molecules in liver IRI, their kinetic profiles have not been characterized and may vary between the different molecules. In this sense, a recent study in renal ischemia/reperfusion has shown that CHOP was significantly activated after 4h and reach a pick after 24 hours of reperfusion [30].

MAPKs are important mediators of stress responses to IRI. They are activated several minutes after reperfusion and have been associated with apoptosis and necrosis [31, 32]. Moreover, prolonged ERS has been linked with the activation of JNK leading to cell death [33]. Also, p38 and ERK inhibition have been shown to protect rat hearts after prolonged hypothermic ischemia [34]. In line with this, it has been shown that p38 and ERK reduction was associated with better fatty liver preservation in IGL-1 solution supplemented with Insulin like growth factor-1 [35]. Along these lines, we found that CA II addition caused a dramatic decrease in ERK, p38 and JNK activation which coincided with the prevention of liver apoptosis, as reflected by the reduction of caspases 3 and 9 and TUNEL-positive cells.

## Conclusions

The addition of CA II to preservation solution (IGL-1) is a promising strategy for improving graft viability. These results confirm, for the first time, the central role of CA II in the prevention of cold IRI in steatotic livers. Further studies are needed to clarify the molecular mechanisms responsible for the liver protection achieved by the addition of CA II to preservation solution.
