# Correction: Carbonic Anhydrase Protects Fatty Liver Grafts against Ischemic Reperfusion Damage

**DOI:** 10.1371/journal.pone.0139411

**Published:** 2015-09-25

**Authors:** Mohamed Bejaoui, Eirini Pantazi, Viviana De Luca, Arnau Panisello, Emma Folch-Puy, Georgina Hotter, Clemente Capasso, Claudiu T. Supuran, Joan Roselló-Catafau

The ninth author’s name is misspelled. The correct name is: Joan Roselló-Catafau.
